# Supplementary material for: Detailed Structure and Pathophysiological Roles of the IgA-Albumin Complex in Multiple Myeloma
Source: Int J Mol Sci. 2021 Feb 10;22(4):1766. doi: 10.3390/ijms22041766 (PMC7916671; doi:10.3390/ijms22041766)
Supplement: Supplementary file 1 [file ijms-22-01766-s001.zip › Supplementary/Supplemental figure1 PDF.pdf]

IgA α chain Fc region
Albumin
Immunoglobulin J chain

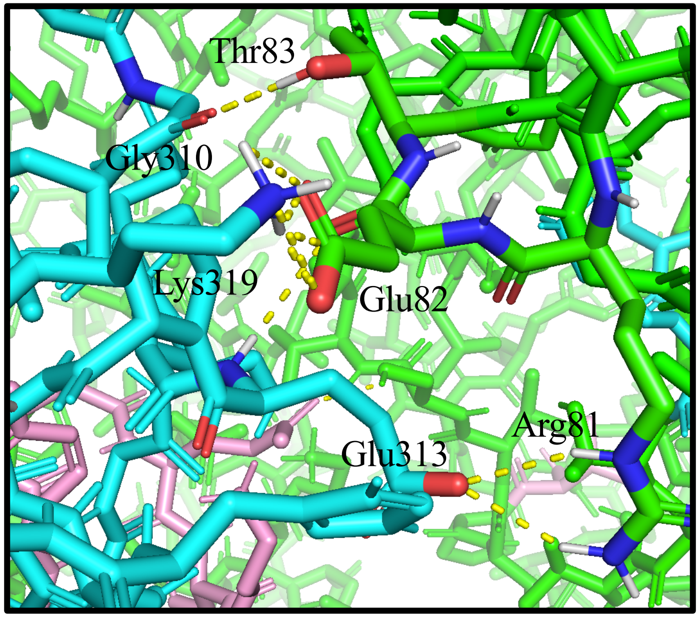

IgA α chain Fc region	Albumin
Lys319	Glu82
Gly310	Thr83
Glu313	Arg81
Arg392	Glu505
Glu393	Lys573
Glu389	Lys500

Supplemental Figure 1. Noncovalent molecular interactions between IgA and albumin
Gray, IgA α chain Fc region; Green, albumin; Purple, immunoglobulin J chain.
Detailed molecular model creation procedures are described in the text.
