# Supplementary material for: Detailed Structure and Pathophysiological Roles of the IgA-Albumin Complex in Multiple Myeloma
Source: Int J Mol Sci. 2021 Feb 10;22(4):1766. doi: 10.3390/ijms22041766 (PMC7916671; doi:10.3390/ijms22041766)
Supplement: Supplementary file 1 [file ijms-22-01766-s001.zip › Supplementary/Supplemental table.docx]

| Protein IDs  **Supplemental Table S1**. Peptides identified from the purified IgA-albumin complex from patient sera. | Gene names | protein name | Sequence | Score |
| --- | --- | --- | --- | --- |
|  |  |  |  |  |
| P02768 | ALB | Serum albumin* | LVAASQAALGL | 239.13 |
|  |  |  | RHPDYSVVLLLR | 194.39 |
|  |  |  | VPQVSTPTLVEVSR | 190.35 |
|  |  |  | DLGEENFK | 186.09 |
|  |  |  | FQNALLVR | 185.42 |
|  |  |  | LVNEVTEFAK | 182.16 |
|  |  |  | LDELRDEGK | 177.70 |
|  |  |  | QTALVELVK | 177.70 |
|  |  |  | KQTALVELVK | 175.65 |
|  |  |  | FKDLGEENFK | 172.27 |
| P01876 | IGHA1 | Ig alpha-1 chain C region | DASGVTFTWTPSSGK | 308.12 |
|  |  |  | QEPSQGTTTFAVTSILR | 297.03 |
|  |  |  | WLQGSQELPR | 198.61 |
|  |  |  | VAAEDWKK | 191.63 |
|  |  |  | TPLTATLSK | 138.06 |
|  |  |  | VAAEDWK | 119.56 |
|  |  |  | SAVQGPPER | 106.88 |
| P01834 | IGKC | Ig kappa chain C region | TVAAPSVFIFPPSDEQLK | 207.97 |
|  |  |  | SGTASVVCLLNNFYPR | 202.09 |
|  |  |  | DSTYSLSSTLTLSK | 189.22 |
|  |  |  | VDNALQSGNSQESVTEQDSK | 173.34 |
|  |  |  | VYACEVTHQGLSSPVTK | 153.97 |
|  |  |  | VDNALQSGNSQESVTEQDSKDSTYSLSSTLTLSK | 148.66 |
|  |  |  | SFNRGEC | 120.62 |
| P01591 | IGJ | Immunoglobulin J chain | CYTAVVPLVYGGETK | 223.08 |
|  |  |  | IIRSSEDPNEDIVER | 186.29 |
|  |  |  | MVETALTPDACYPD | 166.33 |
|  |  |  | KCDPTEVELDNQIVTAT | 155.31 |
|  |  |  | FVYHLSDLCK | 149.82 |
|  |  |  | CDPTEVELDNQIVTATQSNICDEDSATETCYTYDR | 131.69 |
|  |  |  | IIRSSEDPNEDIVERNIR | 101.96 |
| P01009 | SERPINA1 | Alpha-1-antitrypsin | LSITGTYDLK | 233.57 |
|  |  |  | LQHLENELTHDIITK | 191.01 |
|  |  |  | LYHSEAFTVNFGDTEEAKK | 156.29 |
|  |  |  | SASLHLPK | 143.11 |
|  |  |  | LSSWVLLMK | 134.81 |
|  |  |  | FLEDVKK | 133.47 |
|  |  |  | DTEEEDFHVDQVTTVK | 111.07 |
|  |  |  | SVLGQLGITK | 101.38 |

*Proteins in which more than 10 peptides have been detected;10 peptides are listed in descending order of Andromeda score.

**Supplemental Table S2**. Peptides identified from the purified monoclonal IgA from patient sera.

| Protein IDs | Gene names | protein name | Sequence | Score |
| --- | --- | --- | --- | --- |
|  |  |  |  |  |
| P01876 | IGHA1 | Ig alpha-1 chain C region* | DASGVTFTWTPSSGK | 341.35 |
|  |  |  | PTHVNVSVVMAEVDGTCY | 290.10 |
|  |  |  | QEPSQGTTTFAVTSILR | 262.48 |
|  |  |  | DLCGCYSVSSVLPGCAEPWNHGK | 241.41 |
|  |  |  | GDTFSCMVGHEALPLAFTQK | 238.88 |
|  |  |  | TFTCTAAYPESK | 228.84 |
|  |  |  | LAGKPTHVNVSVVMAEVDGTCY | 168.55 |
|  |  |  | KGDTFSCMVGHEALPLAFTQK | 162.42 |
|  |  |  | TFTCTAAYPESKTPLTATLSK | 157.84 |
|  |  |  | NFPPSQDASGDLYTTSSQLTLPATQCLAGK | 122.65 |
| P01834 | IGKC | Ig kappa chain C region | VDNALQSGNSQESVTEQDSK | 455.92 |
|  |  |  | SGTASVVCLLNNFYPR | 307.55 |
|  |  |  | DSTYSLSSTLTLSK | 279.90 |
|  |  |  | TVAAPSVFIFPPSDEQLK | 220.32 |
|  |  |  | VYACEVTHQGLSSPVTK | 209.22 |
|  |  |  | VQWKVDNALQSGNSQESVTEQDSK | 196.11 |
|  |  |  | VDNALQSGNSQESVTEQDSKDSTYSLSSTLTLSK | 143.61 |
| P01024 | C3 | Complement C3 | AYYENSPQQVFSTEFEVK | 264.58 |
|  |  |  | CAEENCFIQK | 239.60 |
|  |  |  | DICEEQVNSLPGSITK | 236.42 |
|  |  |  | DAPDHQELNLDVSLQLPSR | 129.42 |
|  |  |  | DSCVGSLVVK | 125.82 |
|  |  |  | CCEDGMR | 124.37 |
|  |  |  | DFDFVPPVVR | 119.21 |
| P01023 | A2M | Alpha-2-macroglobulin | TEVSSNHVLIYLDK | 244.73 |
|  |  |  | VSVQLEASPAFLAVPVEK | 218.34 |
|  |  |  | VGFYESDVMGR | 213.53 |
|  |  |  | VTAAPQSVCALR | 204.56 |
|  |  |  | VSNQTLSLFFTVLQDVPVR | 120.31 |
| P01009 | SERPINA1 | Alpha-1-antitrypsin | SVLGQLGITK | 246.86 |
|  |  |  | SASLHLPK | 116.90 |
|  |  |  | LSSWVLLMK | 148.04 |
|  |  |  | QINDYVEK | 110.12 |
| P02647 | APOA1 | Apolipoprotein A-I | LLDNWDSVTSTFSK | 297.50 |
|  |  |  | LREQLGPVTQEFWDNLEK | 214.50 |
|  |  |  | LREQLGPVTQEFWDNLEKETEGLR | 173.55 |
|  |  |  | LSPLGEEMR | 123.35 |

*Proteins in which more than 10 peptides have been detected; 10 peptides are listed in descending order of Andromeda score.

| Protein IDs  **Supplemental Table S3**. Peptides identified from the purified albumin from normal sera. | Gene names | protein name | Sequence | Score |
| --- | --- | --- | --- | --- |
|  |  |  |  |  |
| P02768 | ALB | Serum albumin* | ADDKETCFAEEGKK | 321.71 |
|  |  |  | EFNAETFTFHADICTLSEK | 317.91 |
|  |  |  | ALVLIAFAQYLQQCPFEDHVK | 303.04 |
|  |  |  | LVAASQAALGL | 274.55 |
|  |  |  | AAFTECCQAADK | 256.24 |
|  |  |  | MPCAEDYLSVVLNQLCVLHEK | 245.10 |
|  |  |  | LKECCEKPLLEK | 237.53 |
|  |  |  | LVRPEVDVMCTAFHDNEETFLK | 227.57 |
|  |  |  | DVFLGMFLYEYAR | 223.14 |
|  |  |  | ADDKETCFAEEGK | 222.20 |
| P01023 | A2M | Alpha-2-macroglobulin | AAQVTIQSSGTFSSK | 317.99 |
|  |  |  | ALLAYAFALAGNQDK | 259.35 |
|  |  |  | EQAPHCICANGR | 242.35 |
|  |  |  | DMYSFLEDMGLK | 219.50 |
|  |  |  | DTVIKPLLVEPEGLEK | 169.40 |
|  |  |  | AIGYLNTGYQR | 169.09 |
|  |  |  | AYIFIDEAHITQALIWLSQR | 168.45 |
|  |  |  | DNSVHWERPQKPK | 141.89 |
| P02787 | TF | Serotransferrin* | KSASDLTWDNLK | 228.12 |
|  |  |  | CSTSSLLEACTFR | 220.10 |
|  |  |  | CDEWSVNSVGK | 215.72 |
|  |  |  | KPVEEYANCHLAR | 204.32 |
|  |  |  | KDSGFQMNQLR | 201.61 |
|  |  |  | DGAGDVAFVK | 189.70 |
|  |  |  | DDTVCLAK | 169.37 |
|  |  |  | KSCHTAVGR | 145.16 |
|  |  |  | KSCHTGLGR | 143.37 |
|  |  |  | APNHAVVTR | 134.26 |
| P10909 | CLU | Clusterin | EILSVDCSTNNPSQAK | 378.52 |
|  |  |  | ASSIIDELFQDR | 265.77 |
|  |  |  | VTTVASHTSDSDVPSGVTEVVVK | 249.36 |
|  |  |  | LFDSDPITVTVPVEVSR | 233.59 |
|  |  |  | ELDESLQVAER | 194.12 |
|  |  |  | SGSGLVGR | 146.54 |
| P00738 | HP | Haptoglobin | TEGDGVYTLNNEK | 296.65 |
|  |  |  | LRTEGDGVYTLNNEK | 225.72 |
|  |  |  | HYEGSTVPEK | 181.41 |
|  |  |  | HYEGSTVPEKK | 145.90 |
|  |  |  | SPVGVQPILNEHTFCAGMSK | 124.63 |
|  |  |  | DYAEVGR | 107.59 |

*Proteins in which more than 10 peptides have been detected; 10 peptides are listed in descending order of Andromeda score.

**Supplemental Table S4**. Peptides identified from the purified polyclonal IgA from normal sera.

| Protein IDs | Gene names | protein name | Sequence | Score |
| --- | --- | --- | --- | --- |
|  |  |  |  |  |
| P01876 | IGHA1 | Ig alpha-1 chain C region | DASGVTFTWTPSSGK | 322.07 |
|  |  |  | PTHVNVSVVMAEVDGTCY | 280.23 |
|  |  |  | QEPSQGTTTFAVTSILR | 266.77 |
|  |  |  | TFTCTAAYPESK | 242.35 |
|  |  |  | KGDTFSCMVGHEALPLAFTQK | 239.70 |
|  |  |  | GDTFSCMVGHEALPLAFTQK | 226.88 |
|  |  |  | DLCGCYSVSSVLPGCAEPWNHGK | 178.67 |
|  |  |  | LAGKPTHVNVSVVMAEVDGTCY | 171.78 |
|  |  |  | NFPPSQDASGDLYTTSSQLTLPATQCLAGK | 132.94 |
| P01834 | IGKC | Ig kappa chain C region | SGTASVVCLLNNFYPR | 297.83 |
|  |  |  | VYACEVTHQGLSSPVTK | 193.45 |
|  |  |  | VQWKVDNALQSGNSQESVTEQDSK | 188.32 |
|  |  |  | VDNALQSGNSQESVTEQDSKDSTYSLSSTLTLSK | 176.38 |
| P01023 | A2M | Alpha-2-macroglobulin | ETTFNSLLCPSGGEVSEELSLK | 281.49 |
|  |  |  | FSGQLNSHGCFYQQVK | 239.48 |
|  |  |  | EQAPHCICANGR | 236.92 |
|  |  |  | DTVIKPLLVEPEGLEK | 176.56 |
|  |  |  | FQVDNNNR | 158.07 |
|  |  |  | AYIFIDEAHITQALIWLSQR | 150.76 |
|  |  |  | DNSVHWERPQKPK | 118.77 |
|  |  |  | FEVQVTVPK | 114.86 |
| P01009 | SERPINA1 | Alpha-1-antitrypsin | LQHLENELTHDIITK | 243.40 |
|  |  |  | LSITGTYDLK | 179.37 |
|  |  |  | LSSWVLLMK | 171.38 |
|  |  |  | LGMFNIQHCK | 146.79 |
|  |  |  | SASLHLPK | 129.68 |
|  |  |  | SPLFMGK | 114.19 |
|  |  |  | QINDYVEK | 110.12 |
